# Free-floating medial meniscus implant kinematics do not change after simulation of medial open-wedge high tibial osteotomy and notchplasty

**DOI:** 10.1186/s40634-023-00576-1

**Published:** 2023-02-09

**Authors:** Matthias Sukopp, Maoz Shemesh, Elena Pruech, Eran Linder-Ganz, Scott Hacker, Vincenzo Condello, Jonas Schwer, Anita Ignatius, Lutz Dürselen, Andreas Martin Seitz

**Affiliations:** 1 Institute of Orthopedic Research and Biomechanics, Center of Trauma Research Ulm, Ulm University, Medical Center, Helmholtzstrasse 14, 89081 Ulm, Germany; 2 Active Implants LLC, 6060 Primacy Parkway, Suite 460, Memphis, TN USA; 3 Grossmont Orthopedic Medical Group, 5565 Grossmont Center Drive, Building 3, Suite 256, La Mesa, CA USA; 4 Humanitas Castelli Clinic, Via Mazzini, 11, Bergamo, Italy

## Abstract

**Purpose:**

The purpose of this in-vitro study was to examine the kinematics of an artificial, free-floating medial meniscus replacement device under dynamic loading situations and different knee joint states.

**Methods:**

A dynamic knee simulator was used to perform dynamic loading exercises on three neutrally aligned and three 10° valgus aligned (simulating a medial openwedge high tibial osteotomy - MOWHTO) left human cadaveric knee joints. The knee joints were tested in three states (intact, conventional notchplasty, extended notchplasty) while 11 randomised exercises were simulated (jump landing, squatting, tibial rotation and axial ground impacts at 10°, 30° and 60° knee joint flexion) to investigate the knee joint and implant kinematics by means of rigidly attached reflective marker sets and an according motion analysis.

**Results:**

The maximum implant translation relative to the tibial plateau was < 13 mm and the maximum implant rotation was < 19° for all exercises. Both, the notchplasties and the valgus knee alignment did not affect the device kinematics.

**Conclusions:**

The results of the present in-vitro study showed that the non-anchored free-floating device remains within the medial knee joint gap under challenging dynamic loading situations without indicating any luxation tendencies. This also provides initial benchtop evidence that the device offers suitable stability and kinematic behaviour to be considered a potential alternative to meniscus allograft transplantation in combination with an MOWHTO, potentially expanding the patient collective in the future.

## Introduction

Although most meniscus tear patterns are indicated for arthroscopic repair, some require partial or even total meniscectomy [4, 22, 26]. The amount of resected meniscal tissue during such meniscectomy procedures is positively correlated to the risk for the development of knee joint osteoarthritis (OA) [19, 31, 32]. Therefore, artificial scaffold-based replacements [16, 48], three-dimensional (3D)-printed hydrogels [42], allografts [24, 36], autografts [21, 34] and synthetic prostheses including the NUsurface® meniscus implant (Active Implants LLC., Memphis, TN, USA) were developed to overcome the detrimental long-term impact of a meniscectomy on knee joint health [27, 35, 41]. Clinical studies have demonstrated that the NUsurface® device elicits clinical improvements compared to non-surgical treatments [2, 17, 28, 50]. The lateral wall of the non-anchored, free-floating and discoid-shaped device fits within the intercondylar space. Morphological changes of the intercondylar notch are influenced by age and sex differences [18]. Furthermore, the formation of notch osteophytes or a stenotic intercondylar notch is commonly observed in patients with OA [8, 12, 20, 30] and, therefore, occur in candidates that are indicated for the device. These are usually detected during pre-operative imaging and frequently make notchplasty, a widening procedure of the intercondylar notch, a necessary step within the implantation procedure with the aim to reduce the impingement of the device’s lateral wall by achieving a more exact femur-conforming fit [28]. However, due to the free-floating design of the device, an extensive notchplasty might lead to a general instability of the knee joint and, thus, also to device instability leading to an increased risk for luxation [33], compared to the generally low risk for device luxation [47]. Elsner et al. evaluated the long-term performance of the device during the simulation of 5 million gait-like cycles and found no device dislodgement from the joint space [10]. Therefore, the question arises as to whether there is a tissue resection limit to maintain both knee joint and device stability during the normal range of motion (ROM). Consequently, the first aim of this study was to determine the impact of two consecutive notchplasties with increasing tissue removal on the kinematics of the device during different dynamic activities.

Moreover, the importance of limb alignment in knee joint salvage procedures have been described [3, 6, 7, 14]. In the case of congenital or acquired lower limb deformities, the mechanical axis might be altered, which increases the risk for abnormal knee kinematics and concomitant premature joint degeneration [25, 45]. Therefore, operative correction of the mechanical axis prolongates the onset of knee joint OA [5]. The indications for a medial open-wedge high tibial osteotomy (MOWHTO) include varus deformity, mild degeneration, (partial) meniscectomy, knee joint instability and medial compartment overloading [7]. Takeuchi et al. reported an average correction angle of 10° valgus after an MOWHTO [44], leading to an opening of the medial joint space [49] and a significantly reduced tibiofemoral contact load in the medial compartment [1, 38]. Gelber et al. compared in their prospective study on two groups of 30 patients (< 65 years) the functional treatment outcome of a combined OWHTO and partial meniscectomy procedure with a combined OWHTO and partial meniscus replacement procedure and found no short-term patient satisfaction differences between the groups [14]. However, in the case of young (< 40 years), compliant patients who had undergone total meniscectomy and displayed clear pathological signs of mild degeneration states (grade 1 or 2) [6], a combined MOWHTO and meniscus allograft transplantation (MAT) procedure might be indicated [3, 6, 23]. Though currently not included as a use case in the device’s instructions for use, the present biomechanical benchtop study initially assesses the suitability of NUsurface® as an alternative to MAT, by evaluating the impact of an MOWHTO on the device stability: Following an MOWHTO in which the loading of the medial compartment is reduced, the freedom of movement of the femur-conforming device might increase, and with it, there might be a higher luxation risk for the device, particularly in combination with a notchplasty. Therefore, the second aim of this study was to investigate the impact of a 10° shift of knee alignment towards valgus in combination with two consecutive notchplasties on the kinematics of the device during different dynamic activities.

## Methods

### Specimen preparation

**Fig. 1:**
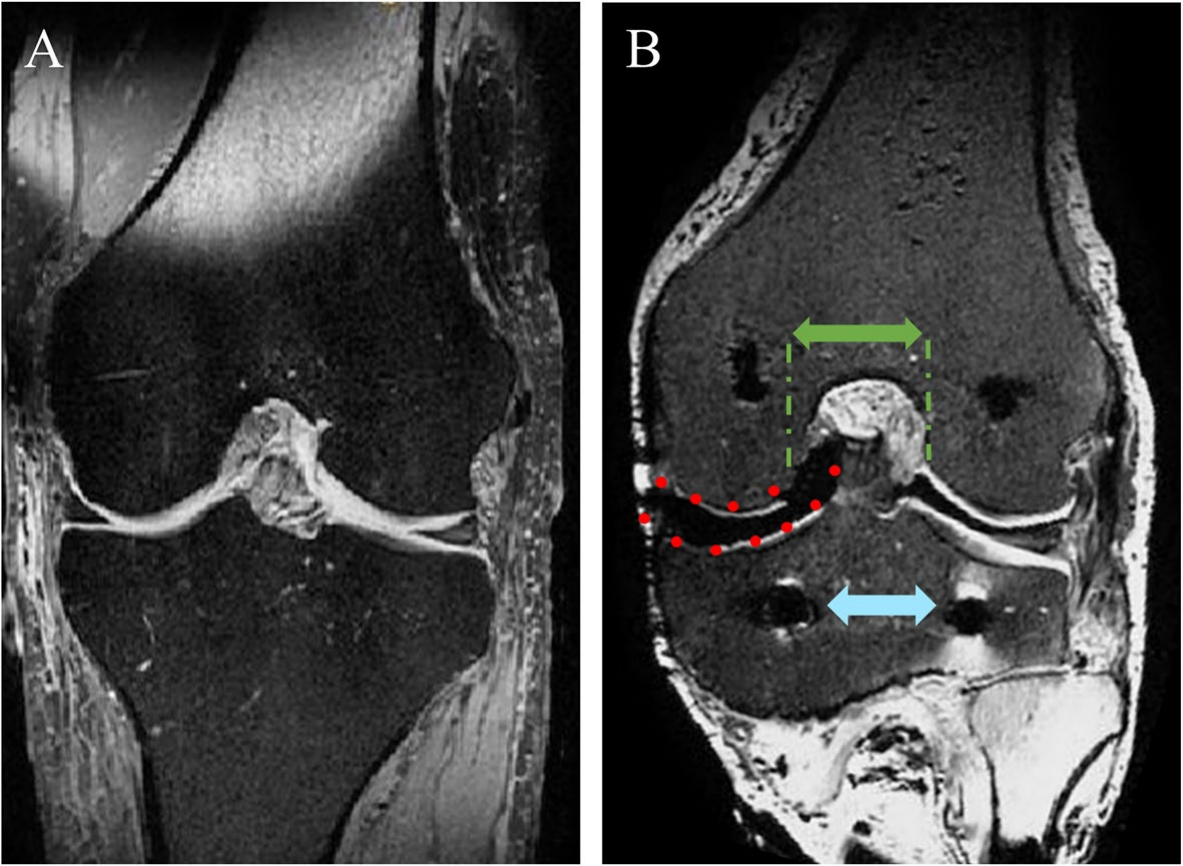
MRI scans in the frontal view (A–P): **A**) intact left knee joint and **B**) resected, with capsuloligamentous structures intact in the left knee joint (white tissue capsule), applied notchplasty (green arrow) with muscle insertion sites (blue arrow, hamstring muscles) and device left in place (red dashed line, black shadow)

**Fig. 2:**
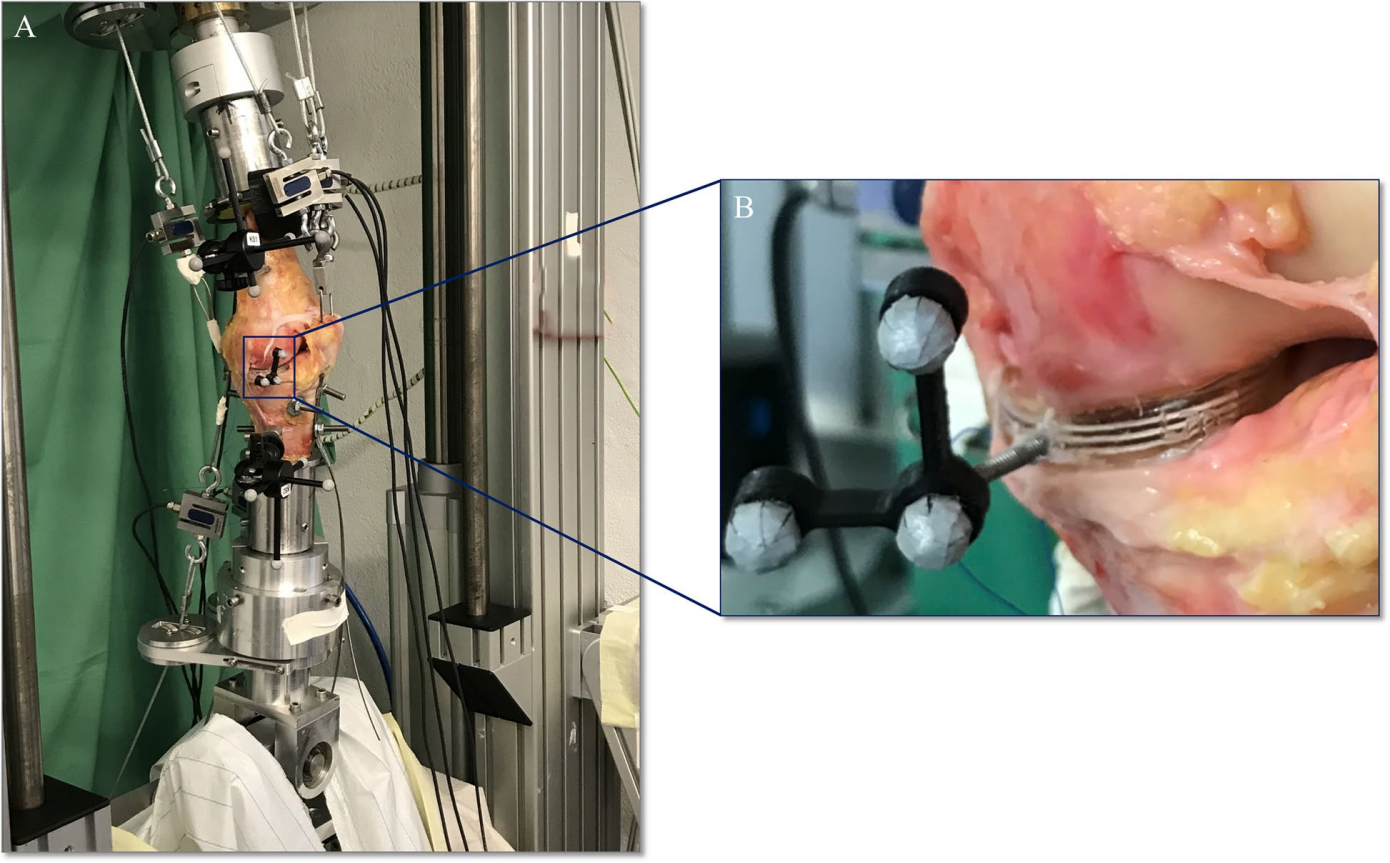
**A** Dynamic knee simulator with embedded specimen, muscle simulation via steel cables and passive reflective rigid marker bodies at the femur, tibia and device. **B** View at the meniscus replacement device, inserted in the medial joint compartment and an attached passive reflective rigid marker body that allows for motion analysis

**Fig. 3:**
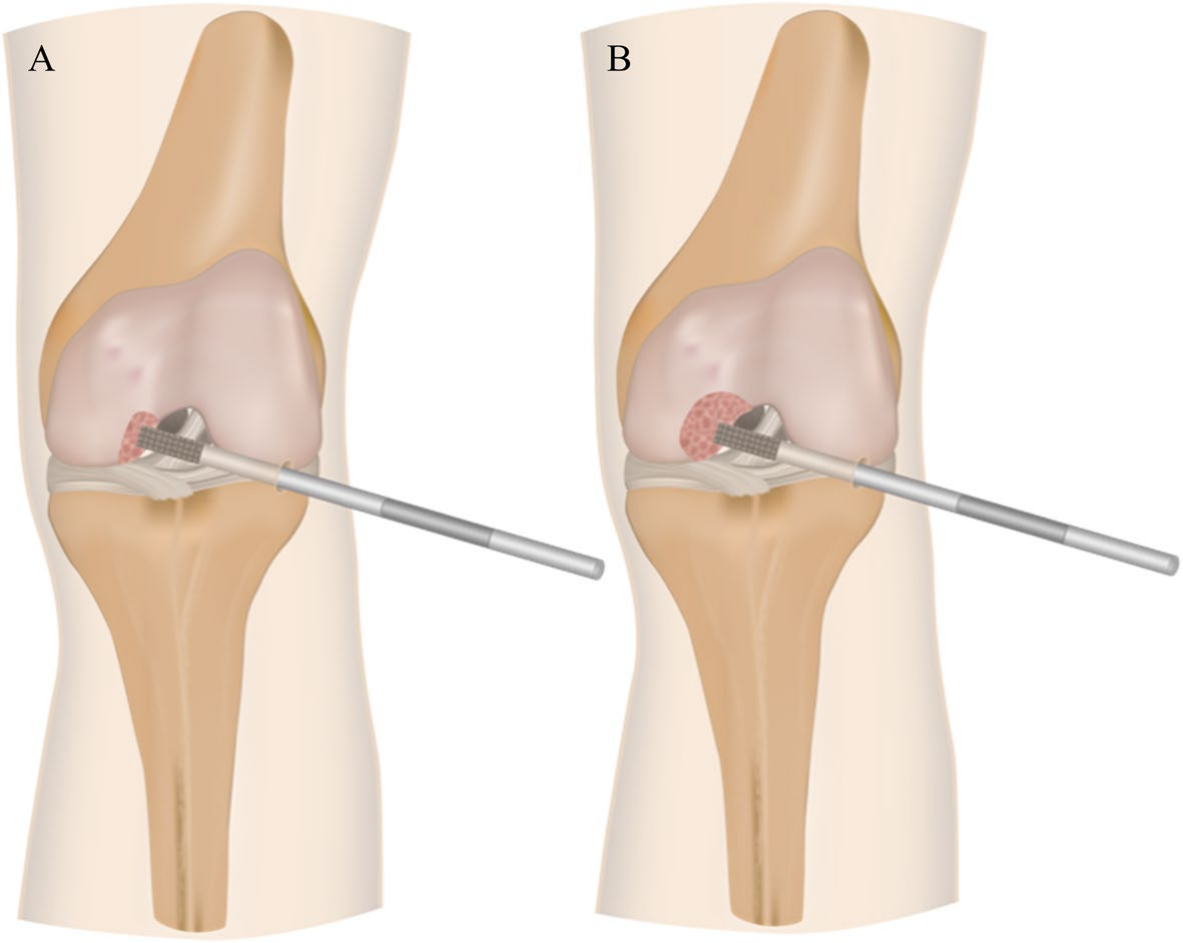
Schematic illustration of the two different notchplasty states applied under arthroscopic surgery in the medial compartment: **A**) Conventional tissue resection (Notch) and **B**) extended tissue removal (xNotch) in the intercondylar notch

Following IRB approval (IRB: 34/19), six fresh-frozen left human knee joints (2 female, 4 male; age: 60.8 ± 7.5 years, BMI: 26.5 ± 3.9) were obtained from an official tissue bank and their soft tissues were resected, leaving the capsuloligamentous structures intact (Fig. 1). Steel cables were firmly connected to bi-cortically anchored pins which were screwed in at the centre of the original muscle insertion sites (Fig. 2-B) to allow simulation of the seven major flexor and extensor knee muscles by utilizing seven pneumatic actuators (Fig. 2-A). Following these initial preparation steps, the knee joints were mounted in an upright position in an Oxford-rig dynamic knee simulator to enhance the further preparation process. Subsequently, two experienced surgeons (SH and VC) performed the arthroscopic NUsurface® device implantation, while starting with a subtotal medial meniscectomy leaving a 2–4 mm meniscal rim intact to provide device confinement. To allow for proper device sizing, a preceding 3D geometrical analysis, based on magnetic resonance imaging (MRI), was performed. The device of the correct size was then inserted into the medial compartment using the designated arthroscopic instruments. The knee joints were tested in three states (Fig. 2): native, conventional notchplasty (Fig. 3-A) and extended notchplasty (Fig. 3-B). Following native testing, the surgeons (SH and VC) performed the consecutive arthroscopic notchplasties, starting with the conventional notchplasty. Following testing, 3D MRI scans of each knee were performed with the device in-situ to evaluate the resected tissue amount during the extended notchplasty. For kinematical evaluation of the knee joint and the relative device kinematics, passive reflective rigid-body-markers were attached to the *antero-medial* aspect of the device (Fig. 2-B) and to the bony landmarks at the femur and tibia (Fig. 2-A). The small device marker was self-developed, in-house 3D-printed and equipped with reflective foil (Scotchlite 7610, 3 M, Minnesota, USA) (Fig. 2, B). The knee joints were kept moist throughout the preparation and testing process using saline solution.

### Biomechanical testing

An Oxford-rig knee simulator, which allowed physiological replication of the whole leg, including the hip joint with an adjustable *caput-collum-diaphyseal* (*CCD*) *angle*, femur, tibia and ankle joint, was used to perform dynamic exercises on cadaveric knee joints [37, 39] while providing all physiological degrees of freedom. Prior to testing, a pilot trial on one additional cadaveric knee joint confirmed that the removal of skin, subcutaneous fat and muscles did not affect the outcome measures.

The specimens were divided into two groups to study the effect of a simulated MOWHTO procedure on device stability: under neutral alignment (*n* = 3) and under 10° alignment shift towards valgus (n = 3) representing the post-MOWHTO state [44]. The alignment shift was achieved by altering the CCD angle by 10°. For each condition, a total of 11 impact exercises were randomly simulated: jump landing, starting at 10° to a flexion angle of 50° at a velocity of 180°/s and back to the initial 10° starting position at a velocity of 120°/s (10°-50°-10°); squatting (10°-70°-10°) at a flexion velocity of 5°/s, axial ground impacts (50 mm) and internal/external tibial impact rotation (± 10°). The axial impacts and tibial rotation were applied at 10°, 30° and 60° knee flexion angles. During the axial impact simulations, the mechanical hip joint remained firmly fixed and ground impacts were applied directly through the simulated mechanical ankle joint. Both, deep knee flexions, as simulated during squatting, and internal/external impacts, which are injury mechanism of meniscus tears, were applied [13, 29].

### Data acquisition

**Fig. 4:**
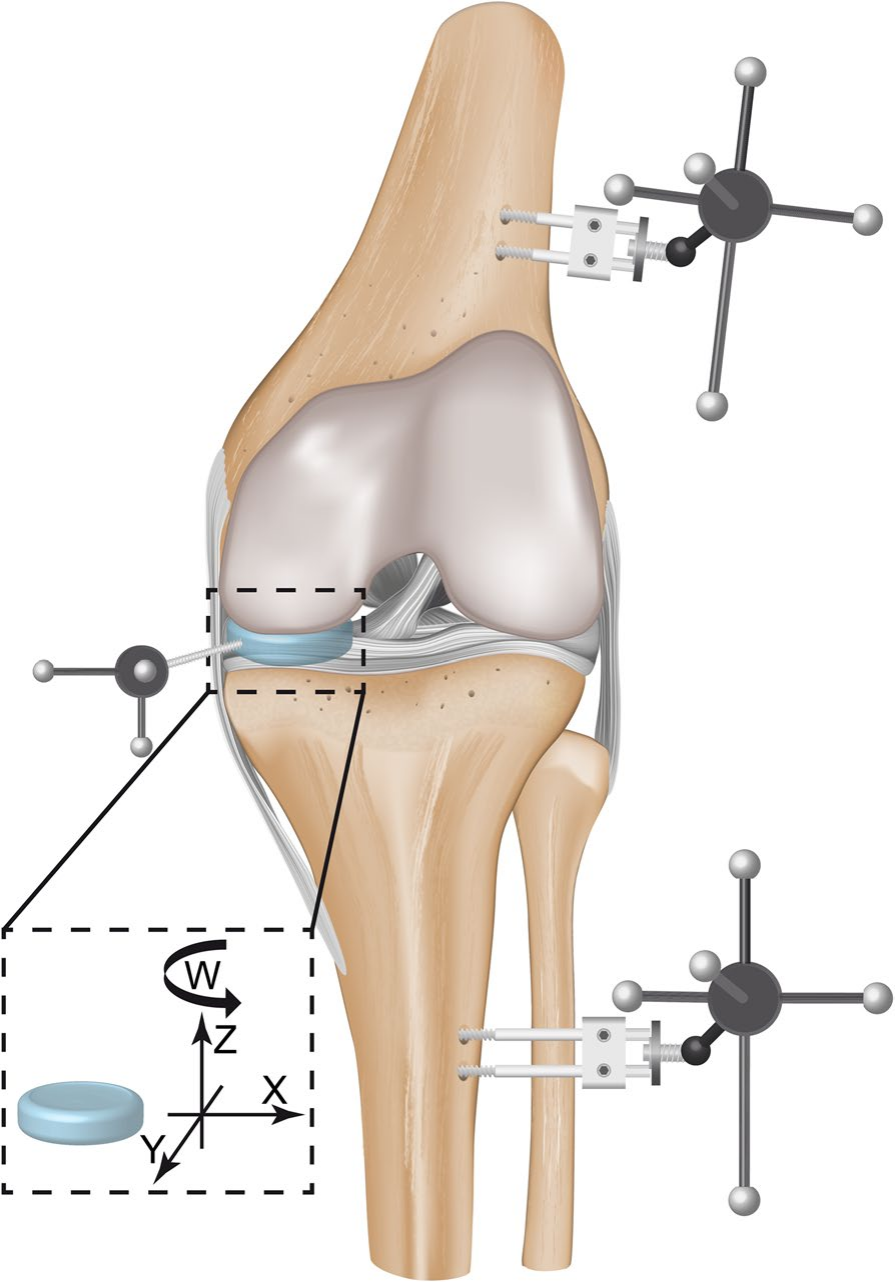
Illustrated frontal view on the knee joint: Coordinate system and the respective three translational axes as well as the rotation W around the corresponding Z axes

Following successful calibration in accordance with the manufacturer’s guidelines and a spatial mean error of 0.13 ± 0.01 mm, a kinematic tracking system (Prime13, OptiTrack, Oregon, USA) with seven cameras was used for motion tracking. To ensure measurement repeatability and accuracy, the calibration procedure was repeated, when the test setup or the specimens were manipulated. The motion sequences were recorded in a specific developer tool (Motive 4.0, OptiTrack) (Fig. 4-B). Export files with kinematic parameters (position, rotation) of each rigid body [15] were generated from the sequences and implemented in a customised analysis protocol (Matlab, The MathWorks, Massachusetts, USA) to determine the maximum spatial device translation and rotation (Fig. 4-A) in relation to the underlying tibial plateau. The description and measurement of six motion components of the rigid bodies were based on Grood and Suntay [15]. The rigid bodies were assumed to be non-deformable. Three translations along the three spatial axes X (*medial-lateral*), *Y* (*anterior-posterior*) and *Z* (*cranial-caudal*) as well as the rotation *W* around the corresponding *Z* axes (rotation of the device in axial tibial rotation) were kinematically evaluated. Therefore, two parameters were assessed to quantify the device kinematics and its stability consecutively rated: 1) absolute translation of the device in relation to the tibial plateau (*device displacement, vectorial addition of X and Y*) and 2) rotation of the device around its axis (*device rotation, rotation around Z*).

### Statistics

**Fig. 5:**
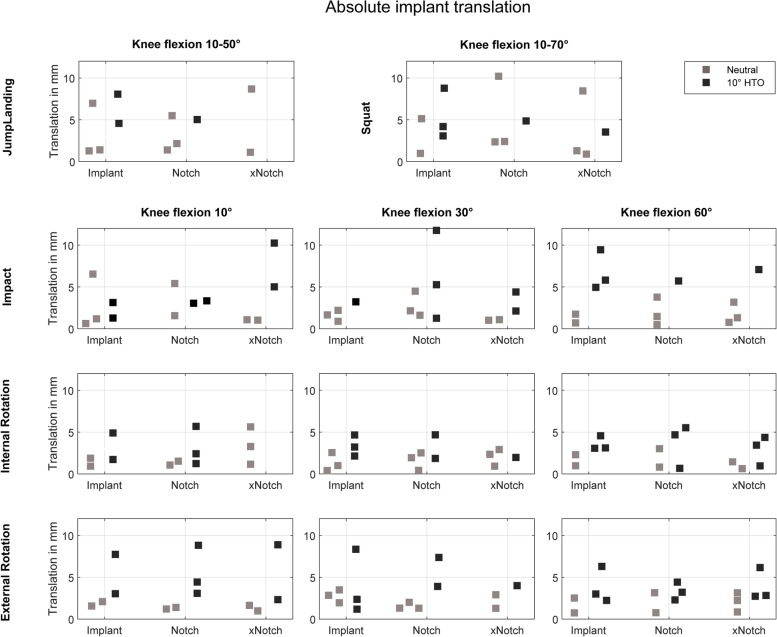
Maximum absolute translation in mm of the device during the four different exercises and three different knee states: intact knee joint (inserted device, no notchplasty), conventional notchplasty and extended notchplasty. Coloured distinction between the neutral leg axis position (grey) and the valgus alignment shift (black)

**Fig. 6:**
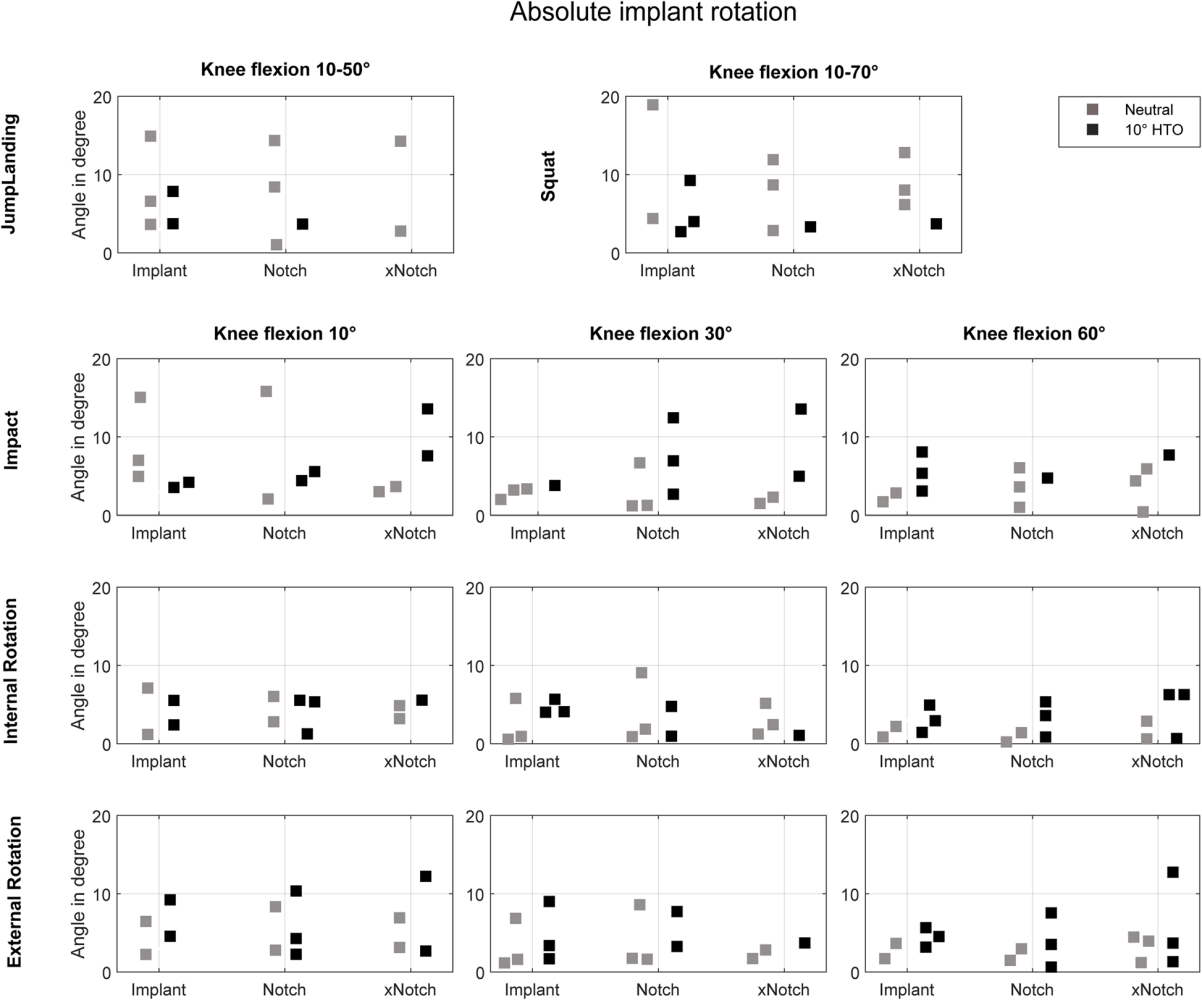
Maximum absolute rotation in ° of the device during the four different exercises and three different knee states: intact knee joint (inserted device, no notchplasty), conventional notchplasty and extended notchplasty. Coloured distinction between the neutral leg axis position (grey) and the valgus alignment shift (black)

Descriptive statistics were applied to present the device kinematics under particular consideration of the limb alignment and the notchplasty states (Figs. 5 and 6). All data is presented in Figs. 5 and 6 where each data point shows the maximum value of the translation or rotation under each test condition. The two groups examined are colour-coded and contrasted in the figures between neutral alignment (grey) and valgus alignment (black).

## Results

### Neutral alignment

In neutral knee alignment, for both, jump landing and squatting the device translation was < 11 mm (Fig. 6) and rotation was < 19° (Fig. 5). Axial ground impact simulation resulted in maximum 6 mm translation and maximum 15° rotation under all three investigated flexion angles (10°, 30°, 60°). The consecutive tissue removal during the notchplasty preparation had no effect on device kinematics during the jump landing, squatting simulation or axial ground impact simulations. During application of 10° external and internal tibial rotation, the device translated < 4 mm (Fig. 5), while rotating < 9° (Fig. 6) under all three knee flexion states with no influence of the notchplasty state on the device kinematics.

### Valgus alignment

During jump landing and squatting simulation with a 10° valgus shift simulating the average post MOWHTO-knee alignment, a maximum device translation of 9 mm (Fig. 5) and rotation of 10° (Fig. 6) were observed. Additionally, under MOWHTO knee alignment, the device kinematics were not altered after tissue resection due to notchplasty preparation during jump landing or squatting. Axial impact simulation resulted in 13 mm translation and 14° rotation, with a slight tendency for a translation increase after notchplasty tissue removal at the 10° knee flexion state. An increased device rotation was observed for progressing notchplasties in all knee flexion states under axial impact simulation. The simulation of internal and external tibial rotation resulted in a maximum device translation of 9 mm and 13° rotation. The device was less stable when applying external tibial rotation (< 9 mm; 13°) compared to internal rotation (< 6 mm; 7°).

## Discussion

The most important finding of this study is that the non-anchored, free-floating device remained in its intended position without luxation under the investigated conditions. The extent of tissue removal during the consecutive notchplasty preparations had no adverse effect on the device. This was also observed in the knee joints with consecutive notchplasties that were investigated following a 10° alignment shift towards valgus, which is similar to a common correction angle after an MOWHTO procedure. To the best of the authors’ knowledge, this is the first study which investigates the influence of two consecutive notchplasty states and different leg alignments on the kinematics of a total meniscus replacement device under dynamic loading simulations.

The results of the present study are in agreement with the range of clinically measured translations [9]. In an open MRI study, translations of up to 10.4 mm of the device during 0° and 120° knee flexion and combined weight-bearing conditions were measured [3], indicating similar translation levels. Furthermore, the device kinematics were close to translation ranges of native medial menisci, where translations of up to 8 mm in a non-weight-bearing setup were measured [46]. The reason for the slightly increased translation of the device will be the prevalence of the high physiological contact forces in our setup. Tissue removal during the notchplasty may play a key role in the assessment of the kinematic device behaviour. Therefore, two expert surgeons who were familiar with the implantation technique performed the notchplasty, comparable to the clinical situation. This notchplasty state was described as the conventional state. Notch stenosis and the formation of notch osteophytes are common corollaries, indicators and even predictors of OA in the knee joint [8, 12, 20, 30]. Therefore, notch stenosis and/or notch osteophytes are expected to occur regularly in suitable candidates for the device. To function as intended, the lateral wall of the device requires adequate room between the intercondylar notch tibial spine and cruciate ligaments. When notch osteophytes articulate against the device, they may accelerate wear formation. A patient’s individual joint condition, such as the location and size of notch osteophytes or the degree of notch stenosis, requires clinicians to make a decision regarding the required degree of notchplasty on a case-by-case basis. This may result in the need for a more extensive notchplasty and concomitantly more tissue removal compared to the conventional state, particularly in the case of a stenotic intracondylar notch. Therefore, two notchplasty states with increasing tissue removal were investigated in the present study to assess their impact on the device kinematics. Furthermore, Gelber et al. and Song et al. have described a positive effect of a combined notchplasty and OWHTO procedure that led to an improved flexion-extension rage recovery which was maintained over time [14, 40], which might be a potential positive side-effect of the notchplasty intervention. The simulated valgus shift in alignment after the MOWHTO induced a joint gap widening on the medial compartment which resulted in a slight translational and rotational increase of the device when compared to neutral leg alignment. This could be mainly attributed to the joint gap widening, and to the concurrently reduced contact pressure on the medial compartment, which appears to move the NUsurface® device more compared to the normal leg alignment conditions [43]. The used knee joint simulator is able to perform dynamic loading situations on cadaveric knee joints that result in physiological tibiofemoral contact pressures [39]. Therefore, the contact pressure between the femur and tibia as well as the resulting contact transmission of the device in the present study could be assumed to be adequate.

This study contributes to existing knowledge of the in-situ biomechanics of the device. Combined knee joint axial loading and internal or external rotation leads to extensive meniscus rotation and according shear stresses in the meniscal body. These shear stresses are likely to result in a meniscal tear and, thus, represent one of the most common injury mechanisms [13, 29]. Similarly, an extensive device rotation would be also likely to result in a device luxation. In this case, the luxation would occur due to a twisting movement and related impingement of the intrinsic geometrical fit of the surface-conforming device. However, critical device behaviour in terms of luxation was not evident from the rotation results of the present study.

### Limitations

This in-vitro study has some limitations. First, the small sample size for each leg alignment group (*n* = 3) allowed only a qualitative and descriptive evaluation. Despite the low number of samples, a post-hoc power analysis indicated a power of 1 − β > 0.4 for the kinematic differences of the device between the intact and extended notchplasty states and 1 − β > 0.3 for the differences between the leg alignment states (G*Power 3.1 [11];; α error = 0.05, n = 3). Therefore, it is assumed that the results of the present study represent promising trends that are likely to be statistically underlined when using a sample size of *n* = 6 (G*Power 3.1; α error = 0.05; power (1 − β) = 0.6) per group. Moreover, it should be mentioned, that despite the low sample size number, we did not see any luxation of the device under the investigated dynamic loading simulations. It should be further noted that a few data points are lacking in the results. The reason for this might be that the optical evaluation led to falsified data due to distortions at the experimental setup and, therefore, it was decided to exclude the respective measurements. Again, also for these simulations where data points had to be removed, the device remained within the joint gap without signs of luxation. Second, the investigated dynamic simulations do not reflect daily activities. However, the simulations and particularly the drop jump and rotation impact sequences represented challenging tasks to provoke a device failure compared to, for example, level walking simulation that includes lower flexion speeds and a reduced total ROM. Third, the MOWHTO was not surgically performed in vitro but simulated by adjusting the leg alignment by an altered CCD angle that resulted in a 10° valgus shift of the knee joint angle. Due to the combination of a slightly compromised stability of the knee joint, which was induced by soft tissue removal, and the high mechanical loads that result in realistic tibiofemoral contact pressures [39] that are applied to the knee joints could have led to lateral hinge fractures after MOWHTO surgery. In addition, it would not have been possible to clearly determine the cause of the obtained results, because the surgical interventions of the MOWHTO and the notchplasty could potentially influence each other. Particularly in contracted knees with a stiff ligamentous situation, it would have been necessary to perform a partial release of the medial collateral ligament to obtain the desired valgisation of the joint line [38], thus also affecting the general kinematics of the knee joint. For these two reasons it was rather decided to perform a kinematic alignment shift instead of a MOWHTO intervention. Future studies are required to prove the device stability under individual patient morphologies to improve the patient outcome by optimising surgical interventions.

## Conclusions

The results of the present in-vitro study show that the non-anchored free-floating device remains within the medial knee joint gap under challenging dynamic loading situations without indicating any luxation tendencies, thereby also providing initial benchtop evidence that the device offers suitable stability and kinematic behaviour to be considered a potential alternative to MAT in combination with MOWHTO, potentially expanding the patient collective in the future.

## Data Availability

The datasets used and/or analysed during the current study are available from the corresponding author on reasonable request.

## Abbreviations

3D: Three dimensional
BMI: Body Mass Index
*CCD*: Caput-Collum-Diaphyseal (angle)
IRB: Institutional Review Board
MAT: Meniscal Allograft Transplantation
MOWHTO: Medial Open-Wedge High Tibial Osteotomy
MRI: Magnetic Resonance Imaging
OA: Osteoarthritis
OWHTO: Open Wedge High Tibial Osteotomy
ROM: Range of Motion
